# Application of three-dimensional printing in plastic surgery: a bibliometric analysis

**DOI:** 10.3389/fsurg.2024.1435955

**Published:** 2024-08-02

**Authors:** Jie Tian, Ming-Jia Jin, Yang Gao

**Affiliations:** ^1^ Department of Thoracic Surgery, West China Hospital, Sichuan University, Chengdu, China; ^2^ Department of Lung Cancer Center, West China Hospital, Sichuan University, Chengdu, China; ^3^ Department of Plastic and Cosmetic Surgery, Shinrong Plastic Surgery Hospital, Chongqing, China

**Keywords:** three-dimensional (3D) printing, plastic surgery, bibliometric analysis, citation analysis, augmented reality

## Abstract

Recent years have seen the publication of numerous papers on the application of three-dimensional (3D) printing in plastic surgery. Despite this growing interest, a comprehensive bibliometric analysis of the field has yet to be conducted. To address this gap, we undertook a bibliometric study to map out the knowledge structure and identify research hotspots related to 3D printing in plastic surgery. We analyzed publications from 1995 to 2024, found in the Web of Science Core Collection (WoSCC), utilizing tools such as VOSviewer, CiteSpace, and the R package “bibliometrix”. Our analysis included 1,057 documents contributed by 5,545 authors from 1,620 organizations across 71 regions, and these were published in 400 journals. We observed a steady growth in annual publications, with Europe, Asia, North America, and Oceania leading in research output. Notably, Shanghai Jiao Tong University emerged as a primary research institution in this domain. The *Journal of Craniofacial Surgery* and *Journal of Oral and Maxillofacial Surgery* have made significant contributions to the field, with Thieringer, Florian M being the most prolific and frequently cited author. Key areas of focus include medical education and surgical procedures, with “3D printing”, “virtual surgical planning” and “reconstructive/orthognathic surgery” highlighted as future research hotspots. Our study provides a detailed bibliometric analysis, revealing the evolution and progress of 3D printing technologies in plastic surgery. As these technologies continue to advance, their impact on clinical practice and patient lives is expected to be profound.

## Introduction

Three-dimensional (3D) printing, also referred to as additive manufacturing or rapid prototyping, enables the swift creation of prototypes or final products through the 3D layering of discrete materials under the meticulous guidance of computer control (1, 2). Having emerged around three decades ago (3), this technology has found progressive applications in specialized medical fields such as cardiac surgery and dentistry (4).

The distinctive feature of medical 3D printing lies in its ability to fabricate precise anatomical structures from volumetric datasets, offering direct visual inspection of human anatomy and pathology (5), thereby becoming increasingly integral in surgical practice and translational research (6). The variable and complex anatomic relationships of craniofacial organs underscore the necessity of a comprehensive understanding of patient anatomy to ensure the safety and success of surgical procedures (7). Recently, there has been a burgeoning interest in leveraging 3D printing and computer software planning in the realm of facial plastic and reconstructive surgery (8). Common materials used in 3D printing for plastic surgery include polylactic acid (PLA), acrylonitrile butadiene styrene (ABS), polyethylene terephthalate glycol (PETG), and polyether ether ketone (PEEK). Additionally, biocompatible materials such as titanium alloys and various polymers are also widely utilized. Different 3D printing methods are employed based on the specific requirements of the surgical application. These methods include fused deposition modeling (FDM), selective laser sintering (SLS), stereolithography (SLA), and multi-jet fusion (MJF). Specifically, various 3D printing techniques used in plastic surgery applications include FDM, which utilizes thermoplastic materials such as PLA, ABS, and PETG to create detailed models layer by layer; SLS, which employs a high-powered laser to sinter powdered materials, forming solid structures; SLA, which uses a laser to cure photopolymer resin layer by layer to produce highly accurate and detailed models; and MJF, which involves the deposition of a binding agent onto a bed of powder material, followed by fusing the material with heat to create complex parts (Supplementary Table S1). This synergy between computer-aided design and manufacturing crafts a detailed surgical model for preoperative planning, including steel plate contouring and the creation of customized patient-specific implants. The advantages of this approach are manifold, encompassing enhanced accuracy of reconstruction, reduction in intraoperative time, diminished metal fatigue, and user convenience (9, 10). The application of 3D printing within plastic surgery has yielded significant advancements in the reconstruction of the mandibular (11), noses, and ears (12, 13), as well as facial skin (14, 15). Furthermore, this technology has also proven instrumental in the domains of resident training, surgical training, and patient education (16, 17).

Bibliometric analysis employs both quantitative and qualitative methodologies to scrutinize the literature within specific research areas of interest. Utilizing visualization technologies, it unveils the structure and distribution of the knowledge graph in the field, highlighting the current research status, hotspots, and emerging trends. Despite the proliferation of papers related to 3D printing in plastic surgery, which has significantly expanded our knowledge base in this area, a comprehensive bibliometric analysis remains absent. Addressing this gap, our study conducts an exhaustive bibliometric analysis to delineate the knowledge structure, direct research clinical questions, and pinpoint study hotspots associated with 3D printing in plastic surgery.

## Materials and methods

### Publication search

**Figure 1:**
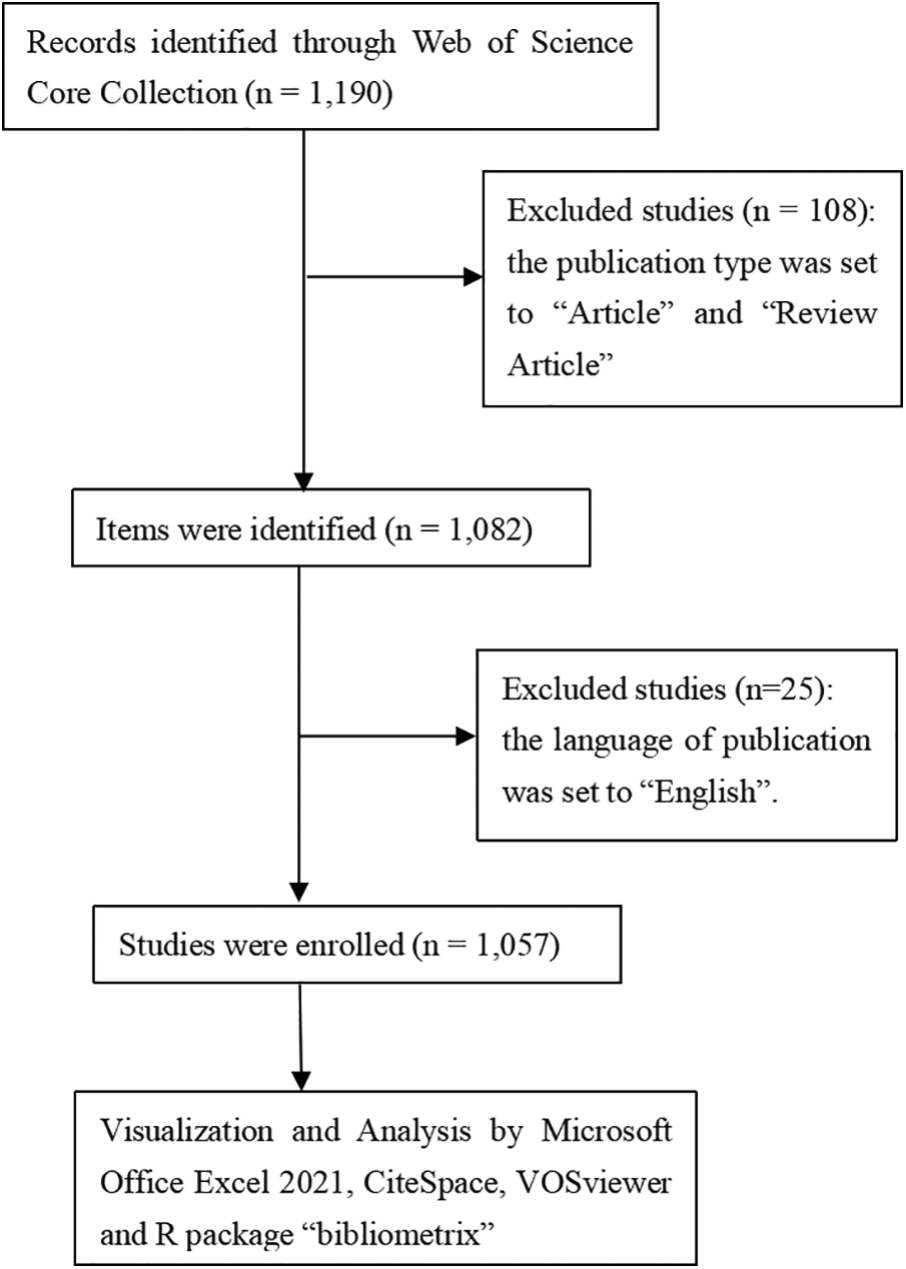
Publications screening flowchart.

Our study conducted a comprehensive search in the Web of Science Core Collection (WoSCC) until 1 July 2024, using keywords such as “{facial plastic surgery} OR {plastic surgery} OR {cosmetic surgery} OR {reconstructive surgery} OR {aesthetic surgery} OR {maxillofacial surgery}” and “{3D printing} OR {Three-dimensional printing} OR {rapid prototyping} OR {stereolithography} OR {additive manufacturing}”. This initial search yielded 1,190 studies. Further refinement was applied by selecting only “Article” and “Review Article” types, leading to 1,082 items. After filtering for English-language papers, 25 studies were excluded, resulting in 1,057 valid studies for analysis (Figure 1).

### Data analysis

For the bibliometric analysis of the identified publications, we employed VOSviewer (version 1.6.19) and CiteSpace (version 6.2 R4) for visualization. VOSviewer was used to create and visualize networks based on data from the literature, revealing the influence of countries, institutions, journals, researchers, and individual publications (18). These networks were designed to reflect citation, co-citation, bibliographic coupling, and co-authorship dynamics. CiteSpace facilitated the construction of dual-map overlays and citation burst analysis (19, 20). Additionally, the “bibliometrix” R package (version 4.3.1) enabled us to examine trending topics and generate global distribution maps of the research (21). Quantitative analysis of the publications was performed using Microsoft Office Excel 2021.

## Results

### Quantitative analysis of publication

**Figure 2:**
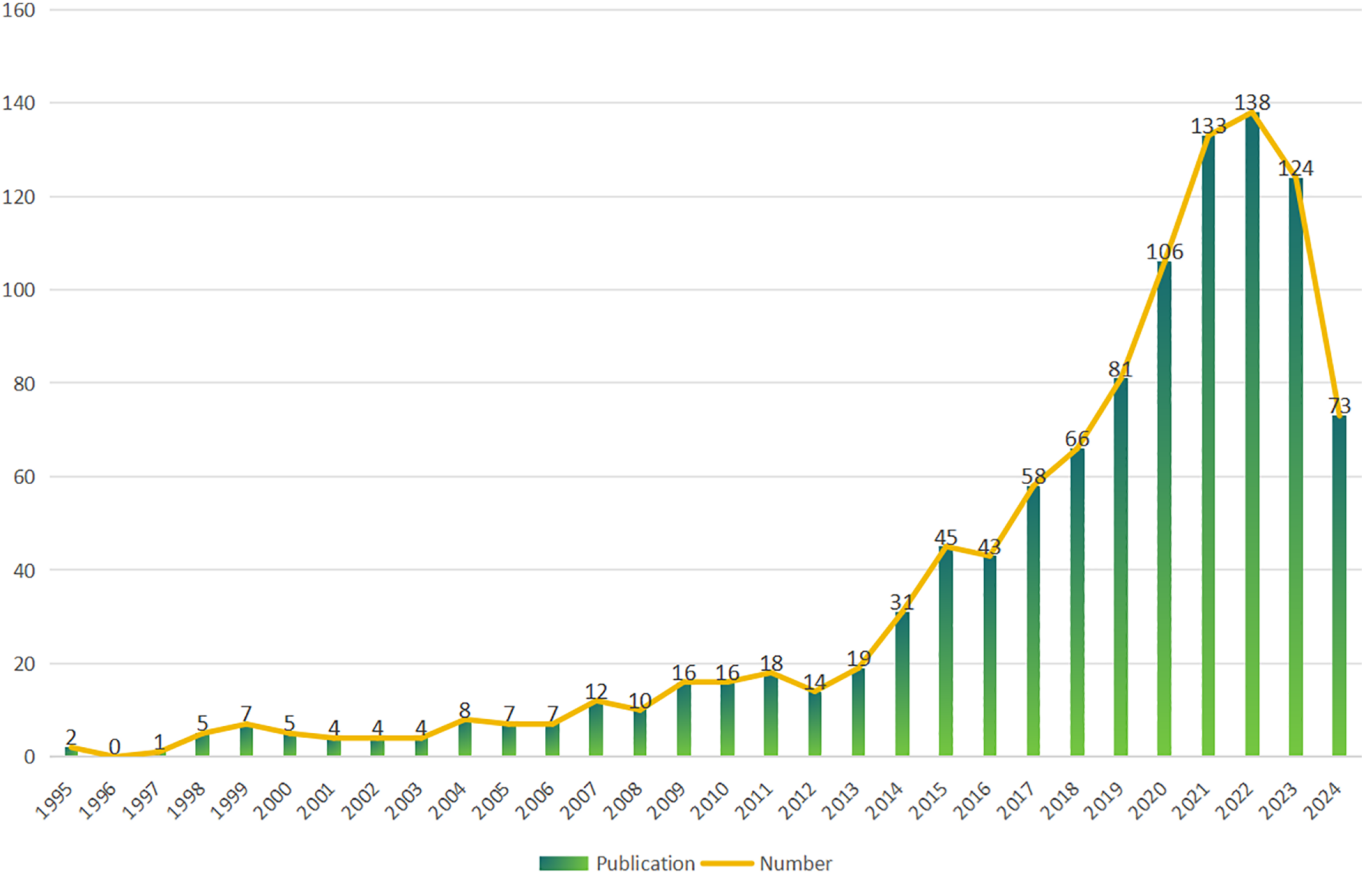
Annual output of research of 3D printing in plastic surgery.

Analysis included 1,057 papers from 1,620 institutions across 71 countries, contributed by 5,545 authors, and published in 400 journals. The temporal span of the publications related to 3D printing in plastic surgery extended from 1995 to 2024 (Figure 2). An initial phase from 1995 to 2013 saw fewer than 20 papers per year; post-2014, a marked increase in publication volume was observed, with an average annual growth rate of approximately 17.27% and an average annual publication count of 82.5.

### Country and institution

**Figure 3:**
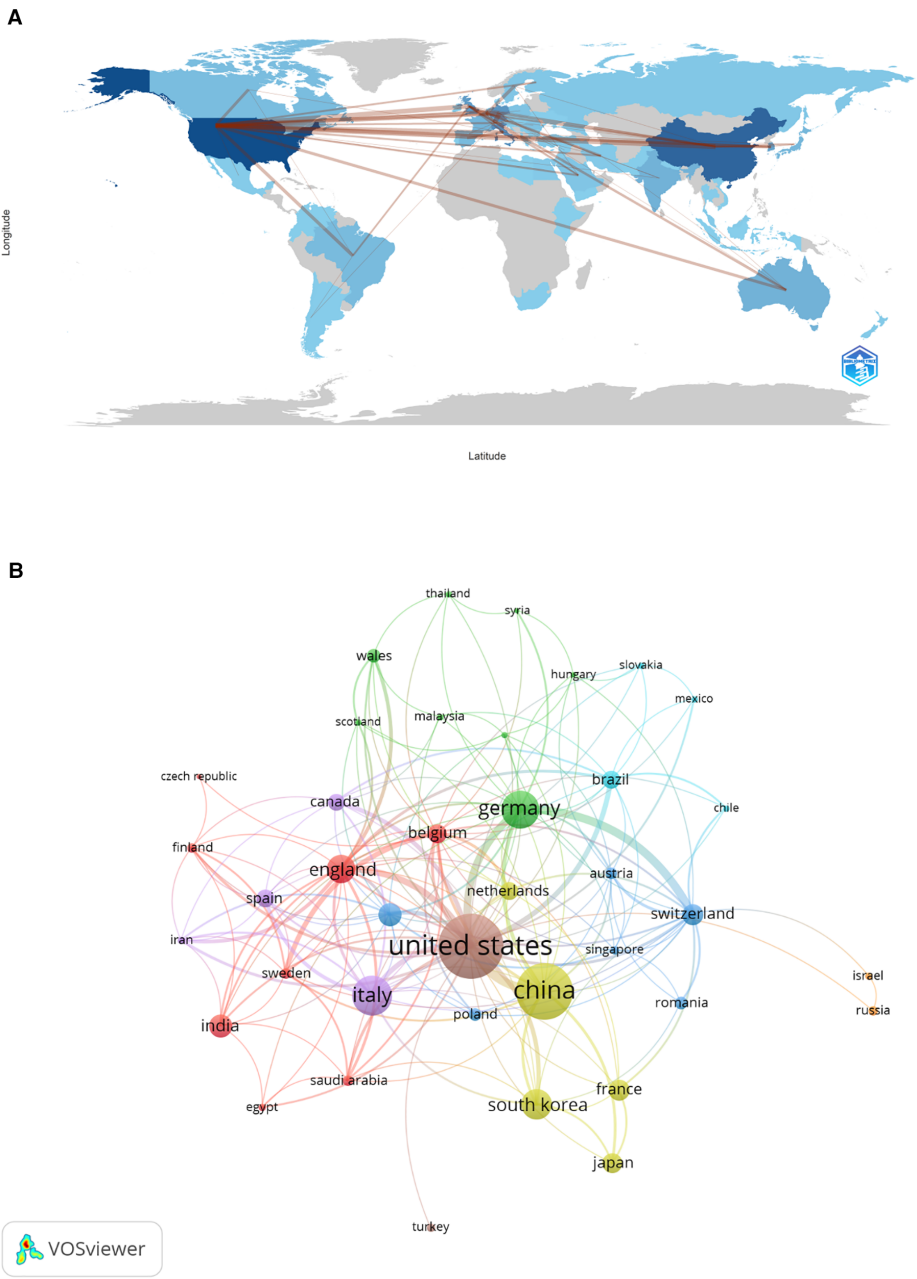
The geographical distribution (**A**) and visualization of countries (**B**) on research of 3D printing in plastic surgery. Minimum number of documents of a country equal to 5, 39 articles met.

**Figure 4:**
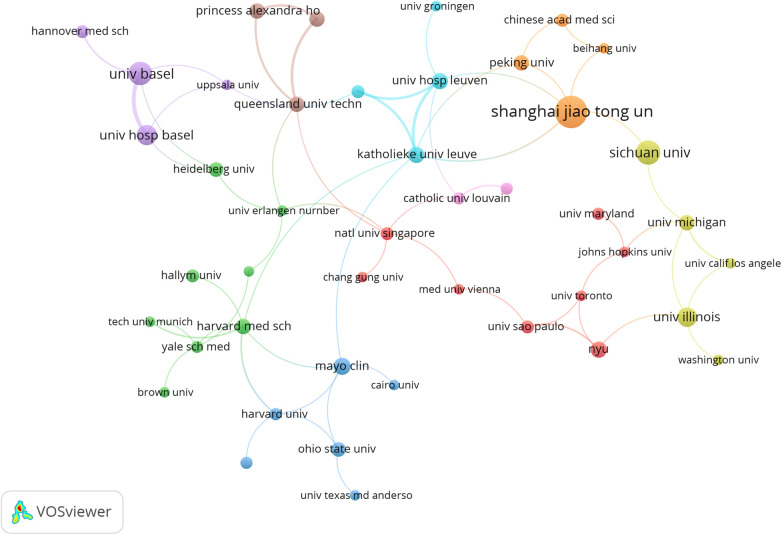
The visualization of institutions on research of 3D printing in plastic surgery. This study selected 44 institutions based on the minimum number of publications equal to 6 for visualization, and constructed a collaborative network based on the number and relationship of publications of each institution.

The surveyed research emanated from 1,620 institutions in 71 countries, highlighting the global interest in 3D printing applications in plastic surgery (Figure 3). The leading contributors were from Europe, Asia, North America, and Oceania, with the United States, China, Italy, Switzerland and Korea being the top five regions. Notably, Shanghai Jiao Tong University, Sichuan University and University of Basel were among the most prolific institutions (Table 1). A collaborative network analysis revealed close collaborations, such as among Shanghai Jiao Tong University, Chinese Academy of Medical Sciences and Peking University; Sichuan University, Shanghai Jiao Tong University, University of Michigan and University of Illinois (Figure 4).

**Table 1:** Top 10 countries and organizations on the research of 3D printing in plastic surgery.

Rank	Country	Counts	Citations	Average citation/publications	Organization	Counts	Citations
1	The United States (North America)	209	5,566	26.63	Shanghai Jiao Tong University (China)	32	943
2	China (Asia)	168	3,244	19.31	Sichuan University (China)	19	316
3	Italy (Europe)	99	1,841	18.60	University of Basel (Switzerland)	18	408
4	Germany (Europe)	90	3,086	34.29	University of Bologna (Italy)	15	200
5	South Korea (Asia)	66	1,331	20.17	University Hospital Basel (Switzerland)	14	307
6	England (Europe)	58	1,678	28.93	University of Illinois (The United States)	13	693
7	Australia (Oceania)	43	1,510	35.12	Yonsei University (Korea)	12	144
8	India (Asia)	43	421	9.79	Alma Mater Studiorum—University of Bologna (Italy)	11	400
9	France (Europe)	37	1,211	32.73	Mayo Clinic (The United States)	11	113
10	Switzerland (Europe)	37	874	23.62	University of Ulsan (Korea)	11	87

### Top journals and co-cited journals

**Figure 5:**
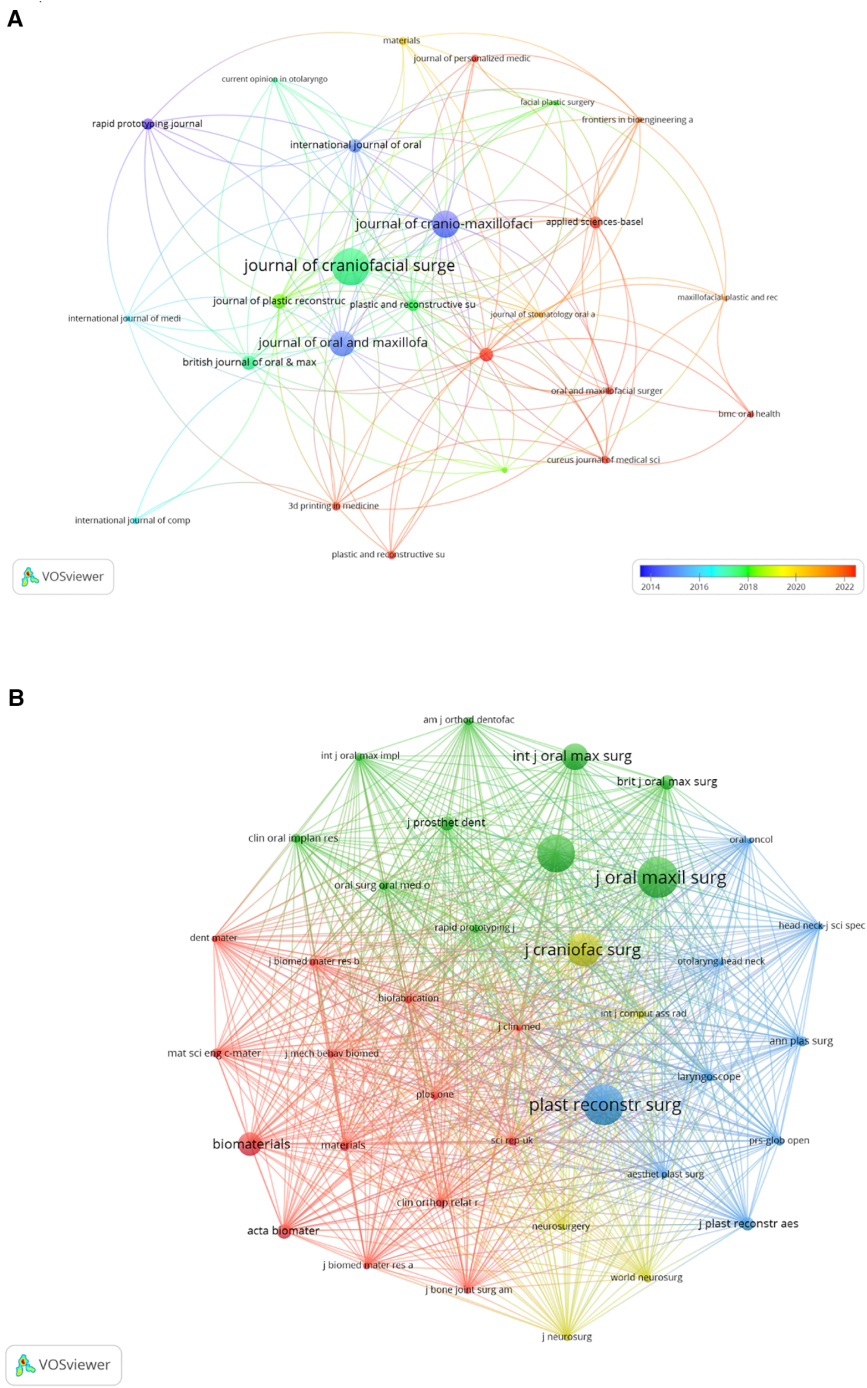
The visualization of journals (**A**) and co-cited journals (**B**) on research of 3D printing in plastic surgery. (**A**) this study enrolled 25 journals based on the minimum number of relevant publications equal to 7 and mapped the journal network. (**B**) More than 130 co-citation journals (38 journals) were filtered to map the co-citation network.

The research was published across 400 journals, with the *Journal of Craniofacial Surgery* leading with the highest number of contributions (*n* = 71) (Table 2). This was followed by the *Journal of Cranio-Maxillofacial Surgery*, *Journal of Oral and Maxillofacial Surgery*, and others. *Rapid Prototyping Journal* emerged as the journal with the highest impact factor (IF = 3.4), followed by the *Plastic and Reconstructive Surgery* and *3D printing in Medicine* (IF = 3.2). A network analysis of the 25 most cited journals revealed significant citation relationships, particularly involving *Journal of Craniofacial Surgery* and other key journals (Figure 5A).

**Table 2:** Top 15 journals and co-cited journals on the research of 3D printing in plastic surgery.

Rank	Journal	Counts	Citations	IF^a^	Q^b^	Co-cited journal	Co-citation	IF^a^	Q^b^
1	Journal of Craniofacial Surgery	71	1,074	1.0	3	Journal of Oral and Maxillofacial Surgery	1,570	2.3	2
2	Journal of Cranio-Maxillofacial Surgery	48	2,066	2.1	2	Plastic and Reconstructive Surgery	1,567	3.2	1
3	Journal of Oral and Maxillofacial Surgery	45	1,399	2.3	2	Journal of Cranio-Maxillofacial Surgery	1,416	2.1	2
4	British Journal of Oral & Maxillofacial Surgery	22	344	1.7	2	Journal of Craniofacial Surgery	1,229	1.0	3
5	Journal of Plastic Reconstructive and Aesthetic Surgery	22	592	2.0	2	International Journal of Oral and Maxillofacial Surgery	892	2.2	2
6	International Journal of Oral and Maxillofacial Surgery	21	939	2.2	2	Biomaterials	744	12.8	1
7	Journal of Clinical Medicine	20	204	3.0	1	British Journal of Oral and Maxillofacial Surgery	417	1.7	2
8	Applied Sciences-Basel	18	58	2.5	2	Acta Biomaterialia	405	9.4	1
9	Plastic and Reconstructive Surgery	17	752	3.2	1	Journal of Prosthetic Dentistry	404	4.3	1
10	Rapid Prototyping Journal	16	454	3.4	1	Journal of Plastic, Reconstructive & Aesthetic Surgery	351	2.0	2
11	3D printing in Medicine	12	127	3.2	1	Materials	254	3.1	1
12	Materials	12	204	3.1	1	Annals of Plastic Surgery	252	1.4	3
13	Plastic and Reconstructive Aurgery-Global Open	12	59	1.5	3	Clinical Oral Implants Research	231	4.8	1
14	Cureus Journal of Medical Science	10	16	1.0	3	Oral Surgery, Oral Medicine, Oral Pathology and Oral Radiology	231	2.0	2
15	Journal of Personalized Medicine	10	67	3.0	1	Materials Science & Engineering C-Materials for Biological Applications	222	8.1	1

^a^ The impact factor of the journal are obtained from Journal Citation Reports 2023.

^b^ The quartile of the journal are obtained from Journal Citation Reports 2023.

**Figure 6:**
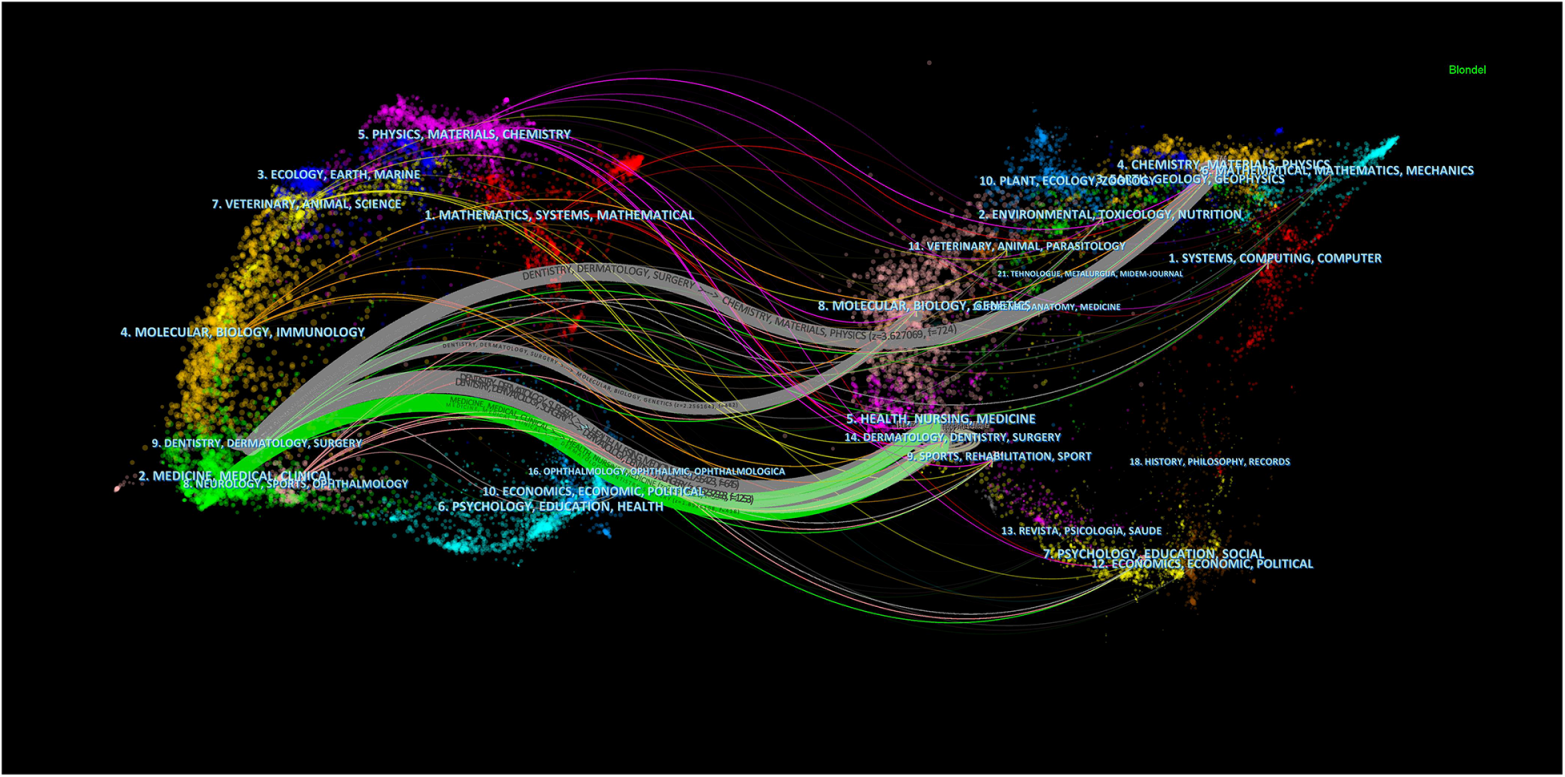
The dual-map overlay of journals on research of 3D printing in plastic surgery.

In the analysis of the top 15 co-cited journals, 26.7% were cited over 1,000 times (Table 2). *Journal of Oral and Maxillofacial Surgery* led with 1,570 co-citations, closely followed by the *Plastic and Reconstructive Surgery* (co-citation = 1,567) and the *Journal of Cranio-Maxillofacial Surgery* (co-citation = 1,416). Furthermore, *Biomaterials* stands out with the highest impact factor (IF = 12.8), with *Acta Biomaterialia* (IF = 9.4) and *Materials Science & Engineering C-Materials for Biological Applications* (IF = 8.1) trailing just behind. We developed a co-citation network from 38 journals (Figure 5B), revealing strong co-cited relationships among them, notably, *Plastic and Reconstructive Surgery* shows significant links with the *Journal of Cranio-Maxillofacial Surgery* AND *Journal of Oral Maxillofacial Surgery*. The dual-map overlay of journals delineates the citation relationships between them (22), with Figure 6 highlighting two major associations: the green path indicates that articles in Health/Nursing/Medicine and Dermatology/Dentistry/Surgery are frequently cited by those in Medicine/Medical/Clinical areas; the gray path shows articles from Chemistry/Materials/Physics, Molecular/Biology/Genetics, Health/Nursing/Medicine, Dermatology/Dentistry/Surgery are predominantly cited by works in Dentistry/Dermatology/Surgery sections.

### Top authors and co-cited authors

**Figure 7:**
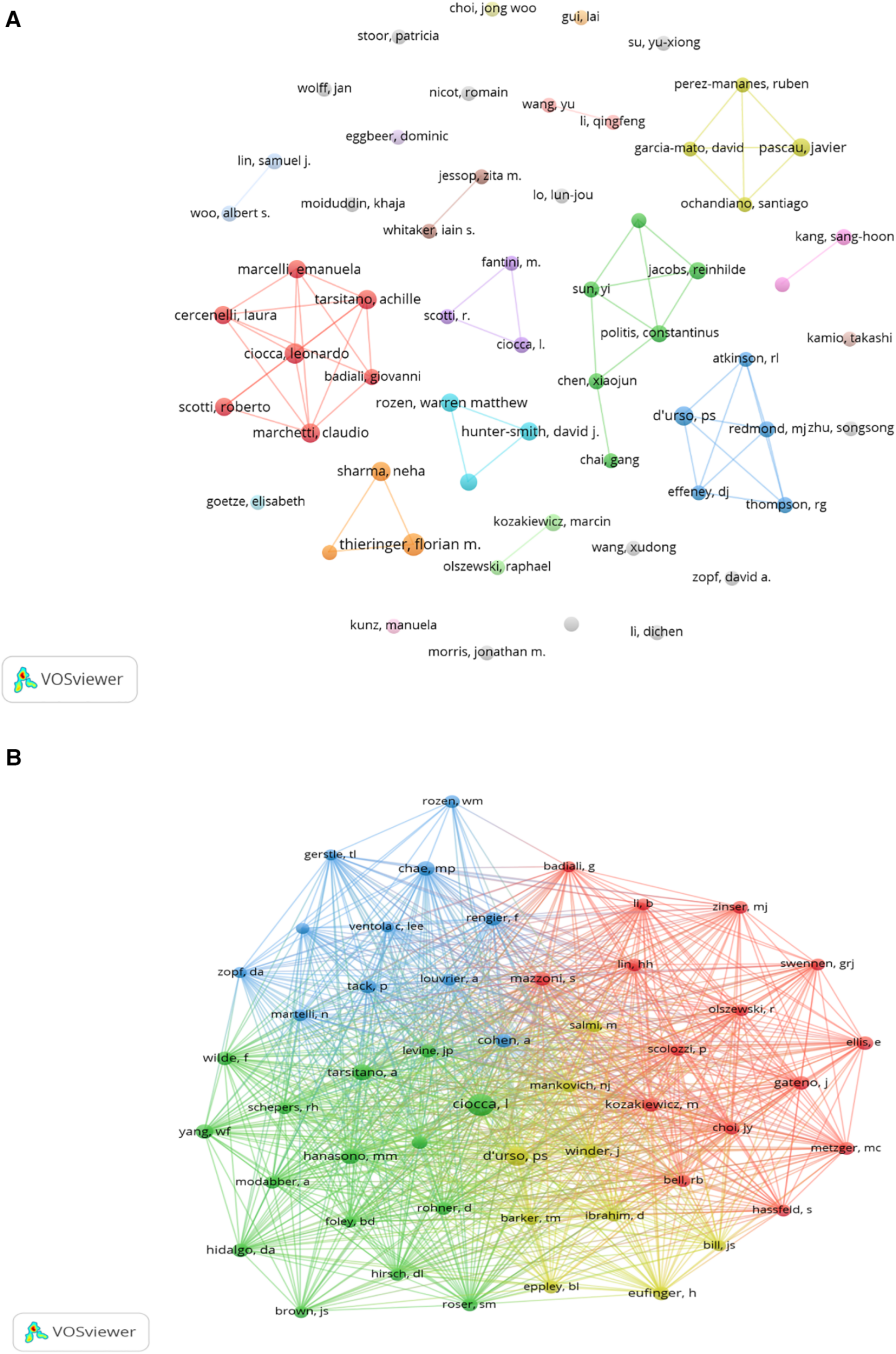
The visualization of authors (**A**) and co-cited authors (**B**) on research of 3D printing in plastic surgery. (**A**) a collaborative network was constructed based on 59 researchers whose number of published documents is more than or equal to 4. (**B**) this study selected 50 authors to map the co-citation network based on minimum co-ciations equals to 30.

Regarding top authors and co-cited authors, our study on 3D printing within plastic surgery includes contributions from 5,545 authors. Notably, 60% of the top 10 authors have published at least eight articles each (Table 3). A collaborative map (Figure 7A) highlights the volume of publications by these researchers, with Thieringer, Florian M leading due to the highest relevance and frequency of publications, followed by Ciocca, Leonardo. Active collaborations were observed among many researchers, including Thieringer, Florian M engagement with Sharma, Neha; Ciocca, Leonardo with Marcelli, Emanuela, Tarsitano, Achille, and others.

**Table 3:** Top 10 authors and co-cited authors on research of 3D printing in plastic surgery.

Rank	Author	Counts	Co-cited Authors	Citations
1	Thieringer, Florian M	12	Ciocca, Leonardo	160
2	Ciocca, Leonardo	9	D'urso, Paul S	114
3	D'urso, Paul S	8	Tarsitano, Achille	80
4	Marchetti, Claudio	8	Hanasono, Matthew M	69
5	Sharma, Neha	8	Yang, Wei-Fa	62
6	Tarsitano, Achille	8	Chae, Michael P	60
7	Cercenelli, Laura	7	Eufinger, Harald	53
8	Hunter-Smith, David J.	7	Wilde, Frank	53
9	Marcelli, Emanuela	7	Hidalgo, DA	52
10	Pascau, Javier	7	Cohen, Adir	51

We observed that 60% of authors were co-cited at least 60 times (Table 3); Ciocca, Leonardo emerges as the most co-cited author (*n* = 160), with D'urso, Paul S (*n* = 114) and Tarsitano, Achille (*n* = 80) also highly cited. A co-citation network of 50 authors (Figure 7B) unveiled extensive collaborations, especially noteworthy among Ciocca, Leonardo, Hanasono, Matthew M and Tarsitano, Achille.

### Top co-cited references

**Figure 8:**
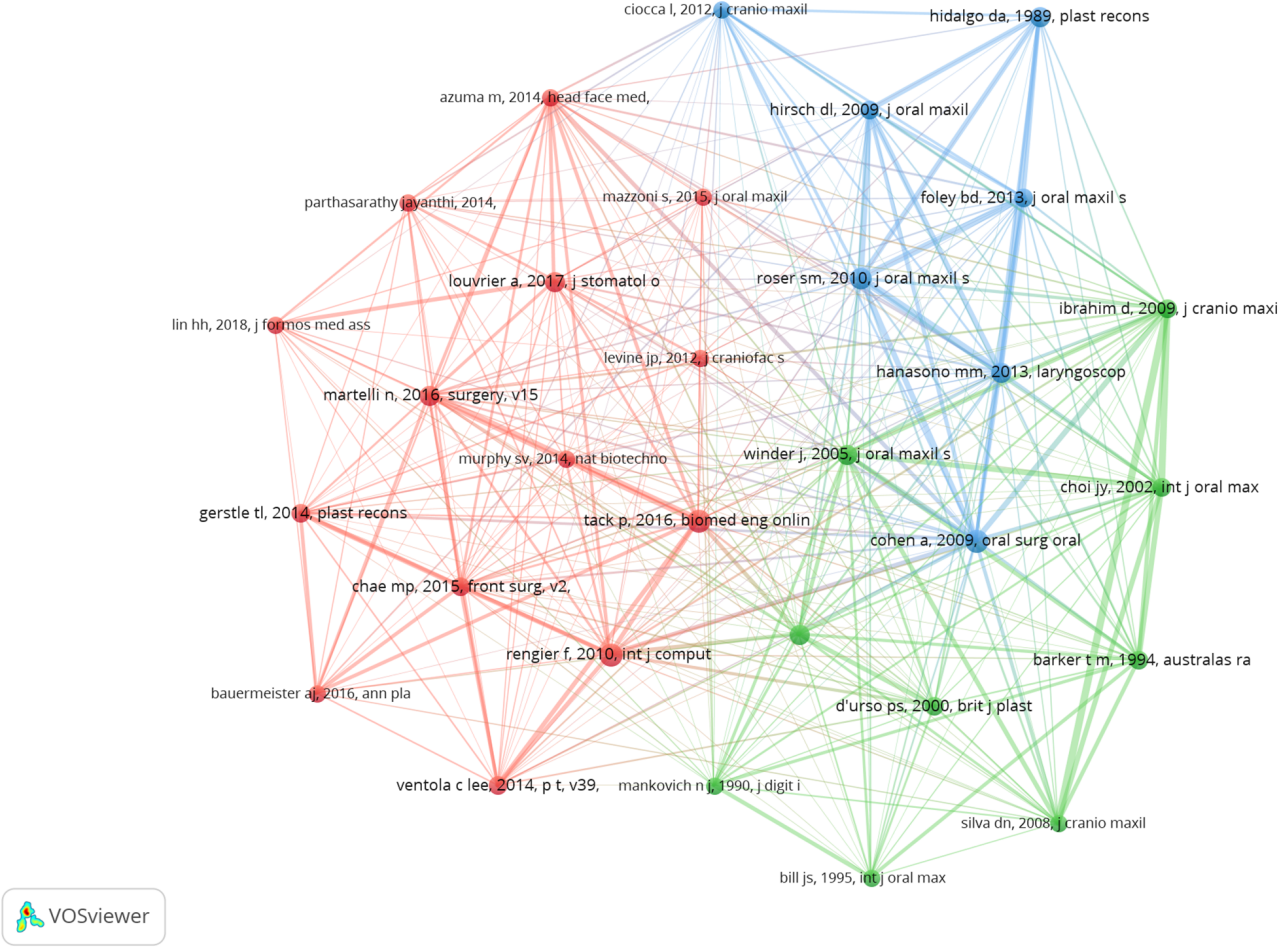
The visualization of co-cited references on research of 3D printing in plastic surgery. a collaborative network was constructed based on 30 references whose number of co-citation is more than or equal to 24.

In our screening of 27,488 co-cited references in the field of 3D printing for plastic surgery, we highlight the top 15 co-cited references, with 86.7% cited more than 30 times (Supplementary Table S2). Total of 30 co-cited references were used to construct the co-citation map (Figure 8). Additionally, Cohen et al. (11) has close co-cited collaborations with Roser et al. (23), Hanasono et al. (24), Hidalgo et al. (25). These articles demonstrate a collaborative effort to document and analyze the impact of 3D printing technology in plastic and reconstructive surgery.

### Reference with citation bursts

**Figure 9:**
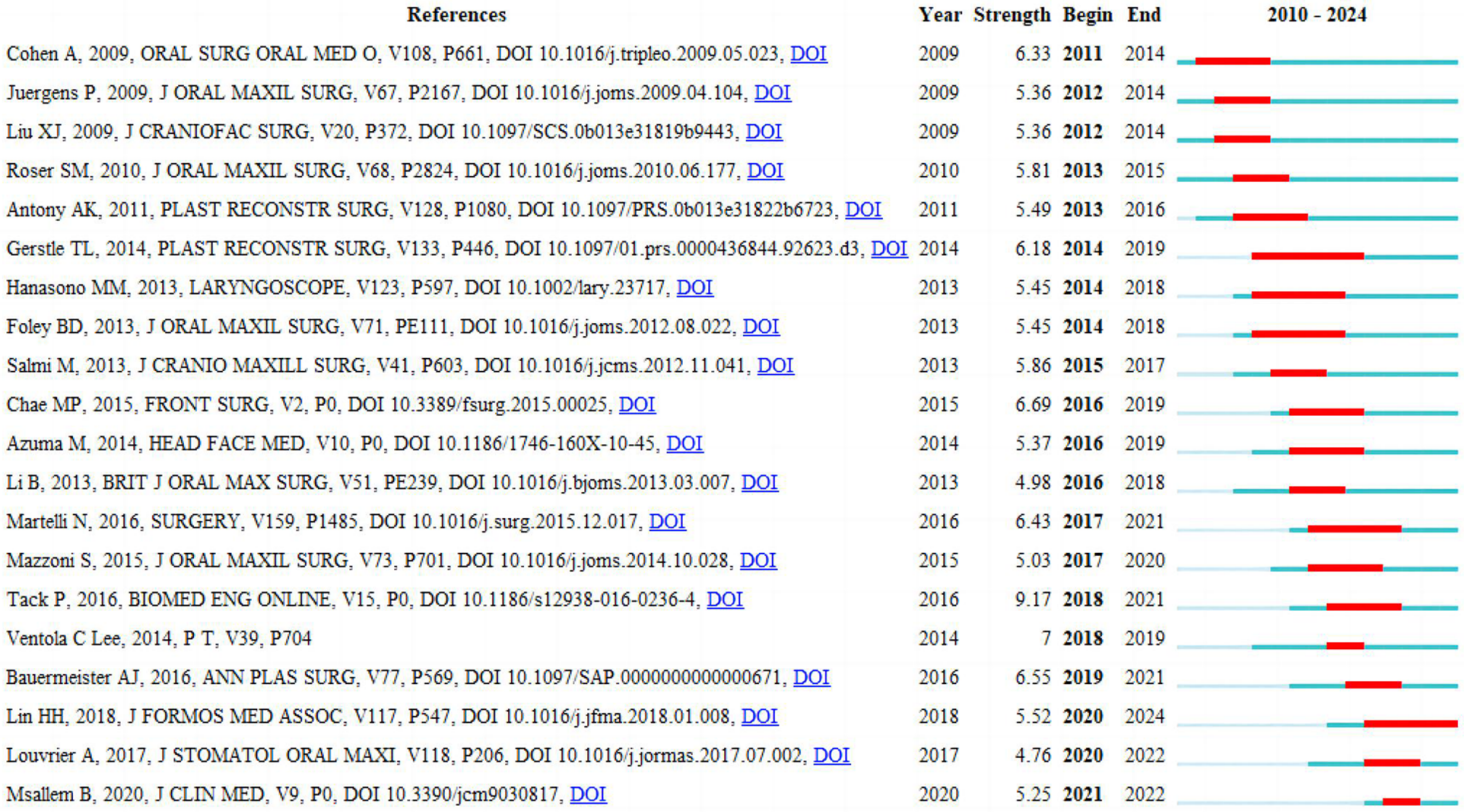
Top 20 references with strong citation bursts. A red bar indicates high citations in that year.

The analysis of citation bursts identified references experiencing rapid citations over specific periods, revealing 20 references with strong citation bursts (red bars) between 2011 and 2024 (Figure 9). This period demonstrated burst strengths ranging from 4.76 to 9.17, lasting between two to six years. We also detailed the core study content from the 20 references with the strongest citation bursts (Supplementary Table S3). The first article had the strongest citation bursts ranging 2011–2014. 3D printing technology offers precise, rapid, and cost-effective mandibular reconstruction, contributing to shorter surgical times. Consequently, this reduces exposure to general anesthesia, minimizes blood loss, and decreases wound exposure time, thereby facilitating a more straightforward surgical process. Moreover, the sixth article had the strongest citation bursts ranging 2014–2019, lasting six years. This article highlights that 3D printing has numerous applications in medicine, including the printing of devices, implants, tissue replacements, and even entire organs. In the near future, plastic surgeons may find this technology indispensable for surgical planning, education, and the design and development of prosthetic devices. Additionally, the eighteenth article had the strongest citation bursts ranging 2020 to 2024, lasting five years. This article highlights the latest applications of 3D printing technology in orthognathic surgery, discussing its impact on treatment feasibility and patient prognosis. Key areas include 3D computer-aided design/computer-aided manufacturing (CAD/CAM), rapid prototyping, additive manufacturing, 3D printed models, surgical occlusal splints, custom guides, templates, and fixation plates. Furthermore, the use of 3D printing methods in orthognathic surgery can achieve optimal functional and aesthetic outcomes, thereby enhancing patient satisfaction.

### Analysis of hotspots and frontiers

**Figure 10:**
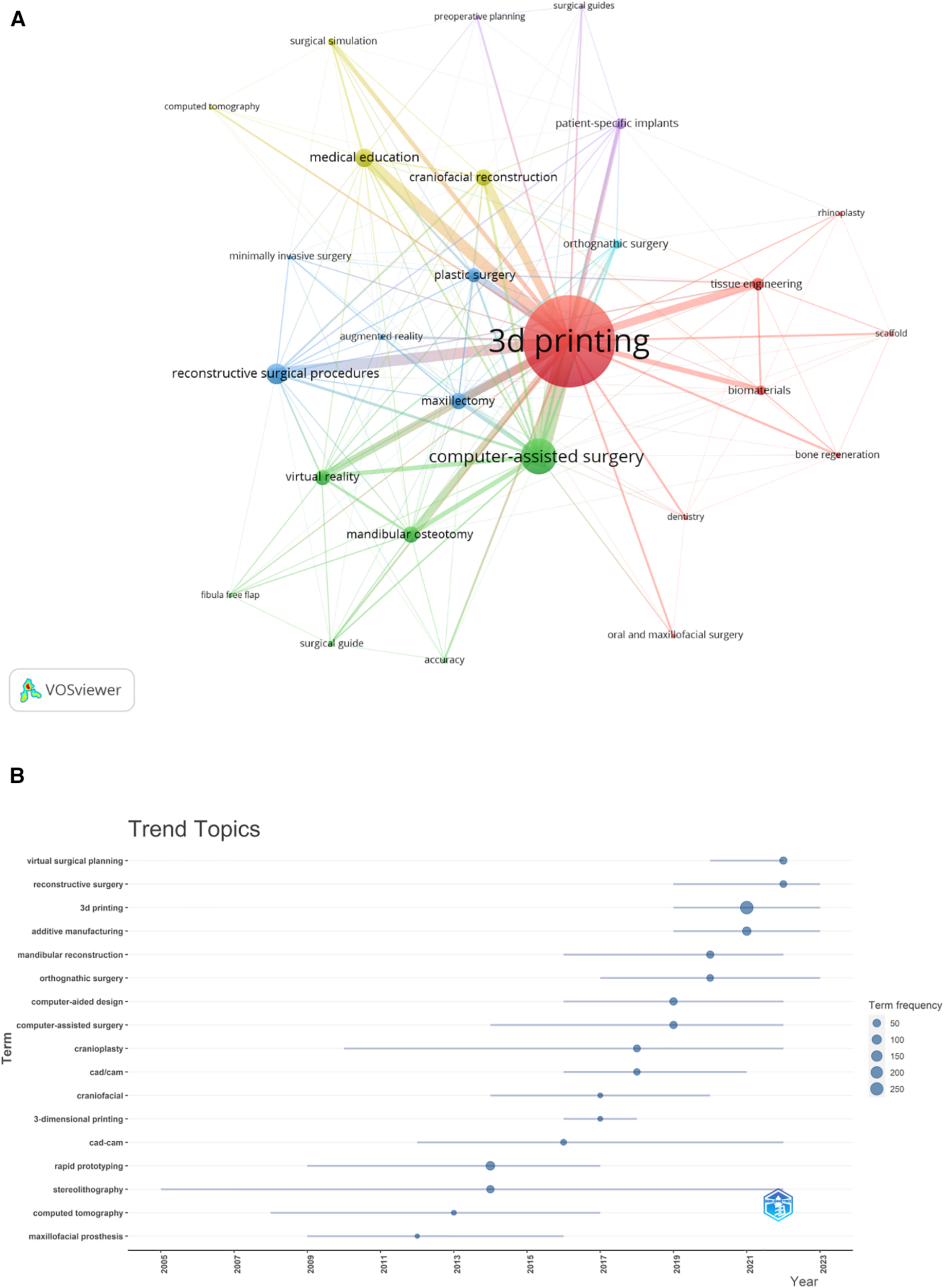
Keyword cluster analysis (**A**) and trend topic analysis (**B**).

In examining hotspots and frontiers, co-occurrence analysis of keywords swiftly identified research focal points within this field (Table 4). The term “3D Printing” appeared 509 times, with nine other keywords surpassing 40 mentions, including computer-assisted surgery, reconstructive surgical procedures, medical education, maxillectomy, craniofacial reconstruction, mandibular osteotomy, virtual reality, plastic surgery and tissue engineering. The network splits into six clusters representing diverse research areas (Figure 10A), with a initial focus on stereolithography and rapid prototyping (Figure 10B). Subsequently, research focus has shifted from early technologies like stereolithography and CT to the application of 3D printing in surgical planning, reconstruction, and education. In recent years, there has been an increasing emphasis on emerging technologies such as bioprinting, virtual surgical planning and reconstructive/orthognathic surgery.

**Table 4:** Top 20 keywords on research of 3D printing in plastic surgery.

Rank	Keywords	Counts	Rank	Keywords	Counts
1	3D Printing	509	11	Patient-Specific Implants	39
2	Computer-Assisted Surgery	162	12	Biomaterials	33
3	Reconstructive Surgical Procedures	81	13	Orthognathic Surgery	31
4	Medical Education	74	14	Surgical Simulation	24
5	Maxillectomy	64	15	Augmented reality	19
6	Craniofacial Reconstruction	62	16	Accuracy	16
7	Mandibular Osteotomy	61	17	Bone Regeneration	16
8	Virtual Reality	59	18	Surgical Guide	16
9	Plastic Surgery	54	19	Minimally Invasive Surgery	15
10	Tissue Engineering	44	20	Oral and Maxillofacial Surgery	15

## Discussion

Plastic surgery, a medical branch dedicated to repairing and reconstructing human tissues, continually explores new technologies to improve surgical outcomes and patients' quality of life. The advent of 3D printing technology not only offers new possibilities for surgical planning and simulation but also pioneers new frontiers in tissue engineering and regenerative medicine through 3D bioprinting. As the technology evolves, 3D printing has become an indispensable tool in plastic surgery. By precisely replicating a patient's anatomical structure, 3D printed models assist surgeons in detailed pre-operative planning and simulation, particularly crucial for complex reconstructive surgeries. In this study, we conducted a comprehensive bibliometric analysis of 3D printing applications in plastic surgery, spanning from 1995 to 2024. While our analysis deepens the understanding of 3D printing' significance in plastic surgery, revealing research structures, hotspots, and trends, it also identifies potential limitations and areas for further exploration.

Research on 3D printing within plastic surgery has garnered contributions from 1,620 institutions across 71 countries, with Europe, Asia, North America, and Oceania leading in scholarly output. Notably, Shanghai Jiao Tong University, Sichuan University and University of Basel have emerged as top contributors. While the collaboration between institutions like Shanghai Jiao Tong University, Chinese Academy of Medical Sciences and Peking University highlights a tight-knit research community. Additionally, Sichuan University ranks among the top in terms of publications, but it has only collaborated with two other institutions. The current landscape of 3D printing research in plastic surgery showcases a diverse and active field, yet it also reveals areas ripe for deeper collaboration. Engaging such institutions in collaborative projects could unlock new insights and methodologies, propelling the field toward innovative solutions and applications in plastic surgery.

This body of work is disseminated across 400 journals, with the *Journal of Craniofacial Surgery* and *Journal of Oral and Maxillofacial Surgery* significantly influencing the discourse on 3D printing in plastic surgery. These journals not only serve as primary platforms for publishing but also play a pivotal role in fostering scholarly connections within the field. The extensive citation and co-citation networks they form underline their centrality to the research ecosystem. Therefore, the role of leading journals in shaping the research dialogue cannot be overstated. Their function as hubs of knowledge exchange makes them critical in identifying emerging trends and fostering interdisciplinary research.

The collective effort of 5,545 authors, with 60% of the top 10 having published at least eight articles each, demonstrates a vibrant and collaborative author community. Thieringer, Florian M and Ciocca, Leonardo are among the most prolific, indicating a core group of researchers driving the field forward. Noteworthy is the observation of significant collaborations, particularly among seven distinct clusters (Figure 7A), highlighting a dynamic interchange of ideas and research strategies. However, the lack of collaboration, as most authors have published independently, suggests room for greater integration and diversity in research endeavors. Therefore, as the field continues to evolve, encouraging a broader spectrum of collaboration among authors, including those who have yet to engage in joint efforts, can significantly contribute to the diversity and depth of research, enhancing the field's capacity to address complex challenges in plastic surgery through 3D printing technologies.

Co-cited references serve as the foundational pillars of a research field. The most cited reference, “Mandibular reconstruction using stereolithographic 3-dimensional printing modeling technology” published in *Oral Surgery, Oral Medicine, Oral Pathology and Oral Radiology* in 2009 (11), highlights that 3D printing technology enables precise, rapid, and cost-effective mandibular reconstruction, shortening surgical times, reducing anesthesia exposure, minimizing blood loss, and decreasing wound exposure, thus simplifying the surgical process. We also found that Cohen et al. (11) has close co-cited collaborations with Roser et al. (23). These articles explore the use of CAD and rapid prototype modeling in mandibular reconstruction. The studies collectively demonstrate that these technologies improve surgical speed, accuracy, and planning. CAD and 3D printing allow for precise preoperative planning and fabrication of surgical models and guides, leading to better alignment of bone segments and reduced operative time. The use of virtual surgical planning further enhances the accuracy of osteotomies and plate positioning. The application of these advanced technologies shows significant benefits in complex mandibular reconstructions, providing improved outcomes and reduced complications. Virtual surgical planning improves accuracy in mandibular defect reconstruction, offering precision that is difficult to achieve through manual placement of the graft, even by experienced surgeons. While surgical cutting guides achieve high precision in mandibular and fibula osteotomies, the ability to manually contour the plate to accurately replicate the plate template remains limited. This limitation reflects in the outcomes, suggesting that further advancements in contouring techniques or automated solutions could enhance the overall effectiveness of the reconstruction process. Additionally, these technologies have shown promise in enhancing patient outcomes, reducing postoperative complications, and increasing overall patient satisfaction. As 3D printing and virtual surgical planning continue to evolve, their integration into routine clinical practice may lead to more predictable and improved results in reconstructive surgery. Future research should focus on overcoming current limitations and exploring new applications of these technologies to further benefit patient care and surgical efficiency.

In analysis of reference with citation bursts (Supplementary Table S3), these articles collectively underscore that 3D printing technology offers precise, rapid, and cost-effective solutions for mandibular reconstruction and other medical applications, enhancing surgical accuracy and efficiency. Virtual surgical planning and CAD combined with rapid prototyping significantly improve outcomes for complex reconstructions. Despite some limitations in manual contouring and high costs, the integration of 3D printing in medical practice is expanding, particularly in surgical planning, education, and the creation of custom implants. Future advancements, including bioprinting and augmented reality, hold promise for further revolutionizing the field, while ongoing research aims to optimize cost-effectiveness and regulatory compliance.

Keyword analysis across publications (Table 4) reveals “3D Printing” as the predominant term, followed by terms related to medical education, plastic surgery, and a spectrum of related surgical and technological themes. The clustering of keywords into distinct categories (Figure 10A) and mainly focused on (i) plastic surgery, maxillectomy, minimally invasive surgery, augmented reality, (ii) medical education, surgical simulation, (iii) patient-specific implants, surgical guides, preoperative planning, (iv) virtual reality, computer-assisted surgery, mandibular osteotomy, (v) tissue engineering, biomaterials, dentistry, oral and maxillofacial surgery, rhinoplasty.

The integration of 3D printing technology into the realm of plastic surgery marks a pivotal shift, heralding a new era of medical innovation. This breakthrough allows for the rapid, precise production of bespoke medical devices and anatomical models, dramatically refining the intricacies of surgical planning and enhancing the predictability of surgical outcomes. Tracing its roots back to 1990, when Mankovich NJ and colleagues first employed stereolithography to fabricate a detailed skull model, the journey of 3D printing has since navigated through the evolution of complex cranio-maxillofacial surgeries (26). These advancements underscore the profound impact of 3D printing in bolstering surgical precision and operational efficiency (27).

Delving deeper into the capabilities of 3D printing, 3D bioprinting emerges as a specialized subset, dedicated to the fabrication of living tissues and organs. This cutting-edge approach leverages living cells and biocompatible materials to craft structures that closely mimic natural biological tissues. The realm of soft tissue reconstruction, encompassing the creation of skin, ears, and noses, has been particularly transformed by this technology. For victims of severe burns, 3D bioprinted skin offers a semblance of normalcy, restoring a natural appearance and feel far beyond the reach of traditional treatment methods (28). Similarly, the fabrication of bones and cartilages through 3D bioprinting paves the way for groundbreaking advancements in facial reconstruction and functional restoration, challenging previous limitations and setting new standards for surgical outcomes (11, 29–32).

The convergence of 3D printing and 3D bioprinting technologies in plastic surgery unveils a dual-pathway approach to medical advancements. On one front, 3D printing excels in elevating surgical precision and streamlining procedures through the creation of personalized models and surgical instruments. On the parallel front, 3D bioprinting ventures into the realm of tissue engineering and regenerative medicine, fabricating tissues and organs that promise to revolutionize patient care. This symbiotic relationship between the two technologies amplifies their potential, broadening the scope of plastic surgery to encompass not only the fundamental aspects of surgical planning and simulation but also the complex, nuanced field of tissue and organ reconstruction. As we stand on the brink of this technological renaissance, the implications of 3D printing and bioprinting in plastic surgery are profound. These innovations not only embody the pinnacle of medical engineering and surgical precision but also herald a future where the limitations of current surgical practices are transcended. Through the lens of 3D printing technologies, plastic surgery is evolving into a discipline characterized by unparalleled accuracy, efficiency, and possibilities for patient rehabilitation and enhancement, truly embodying the transformative power of these advancements.

The introduction of 3D printing into the sphere of surgical training marks a significant transformation in the landscape of medical education, ushering in an era of enhanced surgical competency development. Esteemed for its ability to produce intricate, patient-specific models, 3D printing has become indispensable for procedural training and presents a groundbreaking shift from traditional methods of anatomical study, such as autopsies (16, 17). This innovative approach not only offers an immersive training experience but also plays a crucial role in the skill enhancement and professional growth of emerging plastic surgeons. Through the application of 3D-printed models in preoperative simulations, surgeons can significantly reduce patient risk, improve surgical precision, and bolster their confidence in the operating room (33–36).

Moreover, the rise of augmented reality (AR) and virtual reality (VR) technologies is setting new standards in medical research, particularly enriching the field of plastic surgery with levels of precision and efficiency previously unattainable (37–39). AR and VR are redefining the process of surgical planning, enabling surgeons to visualize and perform procedures with an accuracy and detail that greatly surpass traditional methods. A landmark study by Vles MD, et al., in 2020, showcased the potential of AR and VR to significantly enhance the accuracy of surgical planning, especially in operations requiring intricate anatomical alterations (40). The study highlighted the profound impact of AR and VR in surgical precision, paving the way for these technologies to become cornerstones of surgical education and practice. The integration of AR and VR into surgical training not only promises to elevate the standards of patient care but also to ensure safer, more predictable surgical outcomes.

Expanding upon these technological advancements, the synergy between 3D printing, AR, and VR in surgical training and planning embodies a holistic approach to modern medical education. This fusion not only facilitates a more profound understanding of complex surgical procedures but also enables personalized patient care through meticulous preoperative planning. As these technologies continue to evolve and integrate, they hold the promise of revolutionizing plastic surgery, making procedures safer and more efficient while minimizing potential risks. The future of surgical education and practice is poised on the cusp of a technological revolution, with 3D printing, AR, and VR leading the charge towards a new horizon of medical excellence and patient safety.

While our study presented the applications of 3D printing in facial and cephalic surgery, it is important to acknowledge the significant potential of this technology in other fields such as orthopedics, breast reconstruction, and trauma repair. 3D printing technology has shown significant potential in orthopedics, particularly in the customization of bone implants and surgical guides (41). These customized implants can better match the patient's anatomical structure, improving surgical outcomes and postoperative recovery. For example, 3D-printed hip, knee, and spinal implants have been successfully used in clinical settings (7, 42). Despite its potential, challenges such as the biocompatibility and mechanical properties of materials, as well as the long-term durability of implants, remain. Future research should focus on developing new materials and evaluating the long-term effects of these implants.In terms of breast reconstruction, 3D printing technology is increasingly used to create personalized breast implants or scaffolds. These customized implants provide better aesthetics and feel, while reducing the risk of postoperative complications (43, 44). The main materials used currently include silicone and biocompatible polymers. Future research could explore new biomaterials to enhance the comfort and safety of breast reconstruction implants. Moreover, 3D printing technology is also applied in trauma repair, mainly for customized bone repair and soft tissue regeneration. Personalized repair solutions can be created for trauma patients, such as facial fracture repair and cranial repair (45). Speed and precision are crucial factors in trauma repair.

In addition to 3D printing, other manufacturing techniques such as computer numerical control (CNC) machining and injection molding are used in plastic surgery. CNC machining offers high precision and is suitable for creating complex geometries, but it is typically more expensive and time-consuming compared to 3D printing (46). Injection molding is cost-effective for mass production but lacks the customization capabilities of 3D printing (47). 3D printing technology offers several advantages in plastic surgery, including high precision, the ability to customize implants and surgical guides to match the patient's anatomy, and reduced surgical time. However, there are also disadvantages, such as high costs associated with advanced 3D printers and materials, material limitations, and uncertainties regarding the long-term effects of 3D-printed implants (48). The long-term effects of 3D-printed implants in plastic surgery are still being studied. Key considerations include the biocompatibility and durability of the materials used, as well as potential immune responses. Future research should focus on long-term clinical studies to assess the safety and effectiveness of 3D-printed implants over extended periods (49).

Future research should focus on improving 3D printing speed and accuracy, and developing new materials more suitable for trauma repair. Future studies should expand the scope of 3D printing technology applications in other fields, such as orthopedics, breast reconstruction, and trauma repair, to fully evaluate its potential and effects in different medical areas. Interdisciplinary collaboration between materials science, engineering, and medicine should be encouraged to improve the application level and effectiveness of 3D printing technology. Research should also focus on developing new biocompatible materials, evaluating the long-term effects of implants and repair materials, and increasing the number of clinical trials to validate the effectiveness and safety of 3D printing technology in clinical applications.

This study encountered certain limitations: (i) it relied solely on one database for its search terms, potentially overlooking relevant publications; (ii) it included only English-language papers, possibly missing significant research published in other languages. Future studies can enhance the comprehensiveness of the analysis by addressing data standardization and structure issues. Integrating data from multiple databases, such as Scopus, PubMed, and Google Scholar, will capture a broader spectrum of relevant literature. Additionally, incorporating multilingual databases could provide a more comprehensive analysis. Currently, our analytical software is limited in handling multiple databases and languages due to technical reasons. However, we hope that with future upgrades to the software, we can expand the scope and depth of our research in subsequent phases.

In conclusion, 3D printing technology holds substantial research value and promising applications in plastic surgery, with a noticeable uptrend in annual publications. Europe, Asia, North America, and Oceania are the leading contributors to this field. Journals such as the *Journal of Craniofacial Surgery* and *Journal of Oral and Maxillofacial Surgery* have had a profound influence on the domain. Key areas of focus include medical education and surgical procedures. Furthermore, “3D printing”, “virtual surgical planning” and “reconstructive/orthognathic surgery” emerge as critical keywords indicating future research directions. As these technologies further mature, we expect them to play a greater role in clinical practice to provide patients with better quality treatment options. Future research should delve into the integrated application of these technologies and how to overcome the limitations identified in current studies to drive continued innovation and development in the field of plastic surgery.

## Data Availability

The original contributions presented in the study are included in the article/Supplementary Material, further inquiries can be directed to the corresponding authors.
