# Supplementary material for: Application of three-dimensional printing in plastic surgery: a bibliometric analysis
Source: Front Surg. 2024 Aug 2;11:1435955. doi: 10.3389/fsurg.2024.1435955 (PMC11327138; doi:10.3389/fsurg.2024.1435955)
Supplement: Supplementary file 1 [file Table1.docx]

**Supplementary Table 1: Overview of 3D printing technologies and biomaterials used in plastic surgery.**

| **Printing Technology** | **Biomaterials Used** | **Advantages** | **Applications** |
| --- | --- | --- | --- |
| Fused Deposition Modeling (FDM) | Polylactic Acid (PLA), Acrylonitrile Butadiene Styrene (ABS), Polyethylene Terephthalate Glycol-modified (PETG) | Cost-effective, easy to use, wide material availability | Prototyping, custom implants, surgical guides |
| Selective Laser Sintering (SLS) | Polyamide 12 (PA12), Thermoplastic Polyurethane (TPU) | High strength, good mechanical properties | Orthopedic implants, prosthetics, complex geometries |
| Stereolithography (SLA) | Photopolymer resin | High precision, smooth surface finish | Dental models, hearing aids, surgical models |
| Multi-Jet Fusion (MJF) | Nylon powder | High detail, good mechanical properties, fast production | Functional parts, orthopedic implants, anatomical models |
| Bioprinting | Bioink containing cells and hydrogels | Enables printing of living tissues, high customization | Tissue engineering, organ scaffolds, regenerative medicine |
