# Supplementary material for: Application of three-dimensional printing in plastic surgery: a bibliometric analysis
Source: Front Surg. 2024 Aug 2;11:1435955. doi: 10.3389/fsurg.2024.1435955 (PMC11327138; doi:10.3389/fsurg.2024.1435955)
Supplement: Supplementary file 2 [file Table2.docx]

**Supplementary Table 2: Top 15 co-cited references on research of 3D printing in plastic surgery.**

| Rank | Co-cited reference | Citations | Title | PMID |
| --- | --- | --- | --- | --- |
| 1 | cohen a, 2009, oral surg oral med o, v108, p661, doi:10.1016/j.tripleo.2009.05.023 | 51 | Mandibular reconstruction using stereolithographic 3-dimensional printing modeling technology | 19716728 |
| 2 | tack p, 2016, biomed eng online, v15, doi:10.1186/s12938-016-0236-4 | 49 | 3D-printing techniques in a medical setting: a systematic literature review | 27769304 |
| 3 | rengier f, 2010, int j comput ass rad, v5, p335, doi:10.1007/s11548-010-0476-x | 47 | 3D printing based on imaging data: review of medical applications | 20467825 |
| 4 | roser sm, 2010, j oral maxil surg, v68, p2824, doi:10.1016/j.joms.2010.06.177 | 46 | The accuracy of virtual surgical planning in free fibula mandibular reconstruction: comparison of planned and final results | 20828910 |
| 5 | hanasono mm, 2013, laryngoscope, v123, p597, doi:10.1002/lary.23717 | 37 | Computer-assisted design and rapid prototype modeling in microvascular mandible reconstruction | 23007556 |
| 6 | winder j, 2005, j oral maxil surg, v63, p1006, doi:10.1016/j.joms.2005.03.016 | 37 | Medical rapid prototyping technologies: state of the art and current limitations for application in oral and maxillofacial surgery | 16003630 |
| 7 | hidalgo da, 1989, plast reconstr surg, v84, p71, doi:10.1097/00006534-198907000-00014 | 35 | Fibula free flap: a new method of mandible reconstruction | 2734406 |
| 8 | martelli n, 2016, surgery, v159, p1485, doi:10.1016/j.surg.2015.12.017 | 35 | Advantages and disadvantages of 3-dimensional printing in surgery: A systematic review | 26832986 |
| 9 | d'urso ps, 1999, j cranio maxill surg, v27, p30, doi:10.1016/s1010-5182(99)80007-9 | 34 | Stereolithographic biomodelling in cranio-maxillofacial surgery: a prospective trial | 10188125 |
| 10 | louvrier a, 2017, j stomatol oral maxi, v118, p206, doi:10.1016/j.jormas.2017.07.002 | 34 | How useful is 3D printing in maxillofacial surgery? | 28732777 |
| 11 | chae mp, 2015, front surg, v2, doi:10.3389/fsurg.2015.00025 | 33 | Emerging Applications of Bedside 3D Printing in Plastic Surgery | 26137465 |
| 12 | hirsch dl, 2009, j oral maxil surg, v67, p2115, doi:10.1016/j.joms.2009.02.007 | 33 | Use of computer-aided design and computer-aided manufacturing to produce orthognathically ideal surgical outcomes: a paradigm shift in head and neck reconstruction | 19761905 |
| 13 | ventola c lee, 2014, p t, v39, p704 | 31 | Medical Applications for 3D Printing: Current and Projected Uses | 25336867 |
| 14 | choi jy, 2002, int j oral max surg, v31, p23, doi:10.1054/ijom.2000.0135 | 30 | Analysis of errors in medical rapid prototyping models | 11936396 |
| 15 | foley bd, 2013, j oral maxil surg, v71, pe111, doi:10.1016/j.joms.2012.08.022 | 30 | Mandibular reconstruction using computer-aided design and computer-aided manufacturing: an analysis of surgical results | 23164998 |
