# Supplementary material for: Application of three-dimensional printing in plastic surgery: a bibliometric analysis
Source: Front Surg. 2024 Aug 2;11:1435955. doi: 10.3389/fsurg.2024.1435955 (PMC11327138; doi:10.3389/fsurg.2024.1435955)
Supplement: Supplementary file 3 [file Table3.docx]

**Supplementary Table 3: The main research contents of the 20 references with strong citations bursts**

| Rank | Strength | Main research content | Pubmed ID |
| --- | --- | --- | --- |
| 1 | 6.33 | The 3DP technology provided a precise, fast, and cheap mandibular reconstruction, which aids in shortened operation time (and therefore decreased exposure time to general anesthesia, decreased blood loss, and shorter wound exposure time) and easier surgical procedure. | 19716728 |
| 2 | 5.36 | Computer simulation and rapid prototyping for the reconstruction of the mandible | 19761910 |
| 3 | 5.36 | These results demonstrated that a resin template based on virtual plan and rapid prototyping technique is a reliable messenger to translate from computer modeling to bedside surgical procedures. The repeatability of a virtual plan can be easily and quantitatively evaluated through our three-dimensional differential analysis method. | 19276828 |
| 4 | 5.81 | Virtual surgical planning appears to have a positive impact on the reconstruction of major mandibular defects through the provision of accuracy difficult to achieve through manual placement of the graft, even in the hands of experienced surgeons. Although a reasonably high level of accuracy was achieved in the mandibular and fibula osteotomies through use of the surgical cutting guides, the limited ability to correctly contour the plate by hand to replicate the plate template is reflected in our findings. | 20828910 |
| 5 | 5.49 | The virtual surgical planning technique combined with stereolithographic model-guided osteotomy is the mainstay of the authors' approach to fibular osteotomy when dealing with patients requiring mandibular reconstruction. The authors feel this technology facilitates realization of technical accuracy, aesthetic contour, and functional outcomes and may be particularly useful if free fibular mandibular reconstruction is performed less frequently. | 22030490 |
| 6 | 6.18 | Numerous applications exist in medicine, including the printing of devices, implants, tissue replacements, and even whole organs. Plastic surgeons may likely find this technology indispensable in surgical planning, education, and prosthetic device design and development in the near future. | 24469175 |
| 7 | 5.45 | Computer-assisted design and rapid prototype modeling have the potential to increase the speed and accuracy of mandibular reconstruction. We believe these technologies are particularly useful for cases in which the original architecture of the mandible has been distorted or destroyed. | 23007556 |
| 8 | 5.45 | The use of CAD-CAM (Medical Modeling, Golden, Colorado) technology for the fabrication of surgical resection guides and mandibular reconstruction plates resulted in an accurate surgical result. | 23164998 |
| 9 | 5.86 | A novel measuring technique was developed and its repeatability was found to be good. The accuracy of the PolyJet was higher when compared with SLS or 3DP. | 23333490 |
| 10 | 6.69 | In this review, existing uses of 3D printing in plastic surgery practice spanning the spectrum from templates for facial transplantation surgery through to the formation of bespoke craniofacial implants to optimize post-operative esthetics are described. Furthermore, we discuss the potential of 3D printing to become an essential office-based tool in plastic surgery to assist in preoperative planning, developing intraoperative guidance tools, teaching patients and surgical trainees, and producing patient-specific prosthetics in everyday surgical practice. | 26137465 |
| 11 | 5.37 | Patients with head and neck cancer undergoing reconstructive surgery using a prebent reconstruction plate fabricated according to an MRP mandibular model showed improved mandibular contour compared to patients undergoing conventional mandibular reconstruction. Thus, use of this new technology for mandibular reconstruction results in an improved esthetic outcome with the potential for improved quality of life for patients. | 25338640 |
| 12 | 4.98 | Our CAD/CAM templates provide a reliable method for transfer of maxillary surgical planning, which may be a useful alternative to the intermediate splint technique. Our technique does not require traditional model surgery, scanning of dental casts, or recording of the CAD/CAM splint. | 23566536 |
| 13 | 6.43 | The additional cost and the time needed to produce devices by current 3D technology still limit its widespread use in hospitals. The development of guidelines to improve the reporting of experience with 3D printing in surgery is highly desirable. | 26832986 |
| 14 | 5.03 | These results tend to confirm that the use of CAD-CAM cutting guides and customized titanium plates for upper maxilla repositioning represents a promising method for the accurate reproduction of preoperative virtual planning without the use of surgical splints. | 25622881 |
| 15 | 9.17 | 3D printing is well integrated in surgical practice and research. Applications vary from anatomical models mainly intended for surgical planning to surgical guides and implants. Our research suggests that there are several advantages to 3D-printed applications, but that further research is needed to determine whether the increased intervention costs can be balanced with the observable advantages of this new technology. There is a need for a formal cost-effectiveness analysis. | 27769304 |
| 16 | 7 | 3D printing is expected to revolutionize health care through uses in tissue and organ fabrication; creation of customized prosthetics, implants, and anatomical models; and pharmaceutical research regarding drug dosage forms, delivery, and discovery. | 25336867 |
| 17 | 6.55 | The 3D printing provides the ability to construct complex individualized implants that not only improve patient outcomes but also increase economic feasibility. The technology offers a potential level of accessibility that is paramount for remote and resource-limited locations where health care is most often limited. The 3D printing-based technologies will have an immense impact on the reconstruction of traumatic injuries, facial and limb prosthetic development, as well as advancements in biologic and synthetic implants. | 26678104 |
| 18 | 5.52 | This article highlights the latest applications of 3D printing technology in orthognathic surgery, discussing its impact on treatment feasibility and patient prognosis. Key areas include 3D computer-aided design/computer-aided manufacturing (CAD/CAM), rapid prototyping, additive manufacturing, 3D printed models, surgical occlusal splints, custom guides, templates, and fixation plates. Furthermore, the use of 3D printing methods in orthognathic surgery can achieve optimal functional and aesthetic outcomes, thereby enhancing patient satisfaction. | 29398097 |
| 19 | 4.76 | Patient specific implants (custom-made plates and skeletal reconstruction modules) are much more demanding objects and their manufacturing remains nowadays in the hands of the industry. The main limitation of in-hospital 3D printing is the restrictive regulations applying to medical device. The main limitations of professional 3D printing are the cost and the lead time. 3D printed objects are nowadays easily available in maxillofacial surgery. However, they will never replace a surgeon's skill and should only be considered as useful tools. | 28732777 |
| 20 | 5.25 | All 3D printing technologies create models with satisfactory dimensional accuracy for surgical use. Since satisfactory results in terms of accuracy can be reached with most technologies, the choice should be more strongly based on the printing materials, the intended use, and the overall budget. The simplest printing technology (fused filament fabrication) always scored high and thus is a reliable choice for most purposes. | 32192099 |
